# Incidental uptake of ^18^F-fluorocholine (FCH) in the head or in the neck of patients with prostate cancer

**DOI:** 10.2478/raon-2013-0075

**Published:** 2014-07-10

**Authors:** Marina Hodolic, Virginie Huchet, Sona Balogova, Laure Michaud, Khaldoun Kerrou, Valérie Nataf, Marino Cimitan, Jure Fettich, Jean-Noël Talbot

**Affiliations:** 1 Department for nuclear medicine, University Medical Centre, Ljubljana, Slovenia; 2 Médecine nucléaire, Hôpital Tenon, AP-HP, Paris, France; 3 Comenius University, Bratislava, Slovakia; 4 Université Pierre et Marie Curie, Paris, France; 5 Radiopharmacie, Hôpital Tenon, AP-HP, Paris, France; 6 Nuclear Medicine Unit, National Cancer Institute CRO IRCCS, Aviano, Italy

**Keywords:** FCH, PET/CT, incidentaloma, meningioma, pituitary adenoma, hyperparathyroidism, thyroid adenoma

## Abstract

**Background:**

Positron emission tomography-computed tomography (PET/CT) with ^18^F-fluorocholine (FCH) is routinely performed in patients with prostate cancer. In this clinical context, foci of FCH uptake in the head or in the neck were considered as incidentalomas, except for those suggestive of multiple bone metastases.

**Results:**

In 8 patients the incidental focus corresponded to a benign tumour. The standard of truth was histology in two cases, correlative imaging with MRI in four cases, ^99m^Tc-SestaMIBI scintigraphy, ultrasonography and biochemistry in one case and biochemistry including PTH assay in one case. The final diagnosis of benign tumours consisted in 3 pituitary adenomas, 2 meningiomas, 2 hyperfunctioning parathyroid glands and 1 thyroid adenoma.

Malignancy was proven histologically in 2 other patients: 1 papillary carcinoma of the thyroid and 1 cerebellar metastasis.

**Conclusions:**

To the best of our knowledge, FCH uptake by pituitary adenomas or hyperfunctioning parathyroid glands has never been described previously. We thus discuss whether there might be a future indication for FCH PET/CT when one such tumour is already known or suspected: to detect a residual or recurrent pituitary adenoma after surgery, to guide surgery or radiotherapy of a meningioma or to localise a hyperfunctioning parathyroid gland. In these potential indications, comparative studies with reference PET tracers or with ^99m^Tc-sestaMIBI in case of hyperparathyroidism could be undertaken.

## Introduction

Positron emission tomography-computed tomography (PET/CT) with radiolabeled choline is becoming the first line nuclear medicine examination in patients with prostate cancer^1^, especially where there is evidence of biochemical recurrence.^2^ ^11^C-choline has low urinary excretion, which is favourable for detecting pelvic foci, but its routine use is not possible in centres lacking an on-site cyclotron. ^18^F-fluorocholine (FCH), which can be delivered as easily as ^18^F-fluorodeoxyglucose (FDG), has proven clinical utility for PET imaging of cancers with slow growth and low aggressiveness, frequently missed with FDG.^3^

FCH is currently registered in several EU countries for the detection of bone metastasis in prostate cancer, which is currently its most frequent indication for use. FCH foci can reveal secondary lesions of prostate cancer not only in the skeleton but also in soft tissue, and may be found in unexpected locations such as penis.^4^ But FCH foci may also correspond to other primary cancers^3^ or inflammatory lesions.^5^

Foci in the head or in the neck are unexpected in patients referred for prostate cancer.^6^ We reviewed the reports of FCH PET/CTs performed in our centres in patients with prostate cancer, to select those in which such foci have been reported. We then searched whether the origin and the nature of each focus has been characterised during follow-up. As FCH PET/CT is developing rapidly, we consider it is useful to share experience about the frequency of incidental FCH foci in the head or in the neck, about their possible benign non-inflammatory aetiology and to speculate on a potential indication of FCH PET/CT in the management of those tumours, in comparison with other PET tracers according to a review of literature.

## Patients and methods

The patients, referred for prostate cancer staging or restaging, were fasting for 6–10 hours prior to FCH PET/CT. PET/CT was performed after intravenous injection of 200–300 MBq of FCH (IASOcholine^®^, Graz, Austria, or Advanced Accelerator Applications, Saint Genis-Pouilly, France), according to the body weight of the patient. Whole body acquisition was performed during two minutes for each of 9-10 bed positions, from midthigh to skull, using Siemens Biograph mCT or Philips TF16 PET/CT scanners. Whole body images were presented in the usual transaxial, coronal and saggital slices, for PET, CT and PET/CT fusion.

In University Medical Centre Ljubljana, the reports of FCH PET/CTs performed in prostate cancer patients were reviewed from 29^th^ February 2012 until 11^th^ November 2012. FCH PET/CTs were performed in compliance with Slovenian marketing authorisation granted to Iasocholine in April 2011.

In Hospital Tenon, in Paris, the reports of FCH PET/CTs performed in prostate cancer patients were reviewed from 12^th^ November 2004 until 11^th^ November 2012. FCH PET/CTs were performed as part of two successive clinical studies (CH02 Eudra CT number: 2004-003019-21 and then Ichorpro EudraCT number 2007-004419-69) until November 2009 and then in compliance with the French marketing authorisation granted to Iasocholine in April 2010.

We searched for solitary or multiple lesion(s) in the head (including brain) or in the neck, excluding only images evocative of bone metastatic spread or non-focal uptake such as diffuse intense uptake by the thyroid gland or the physiologic FCH uptake by the salivary or the lachrymal glands. When such foci were visible in the head or in the neck, we requested further data in order to try to characterise the lesions.

## Results

**FIGURE 1.**
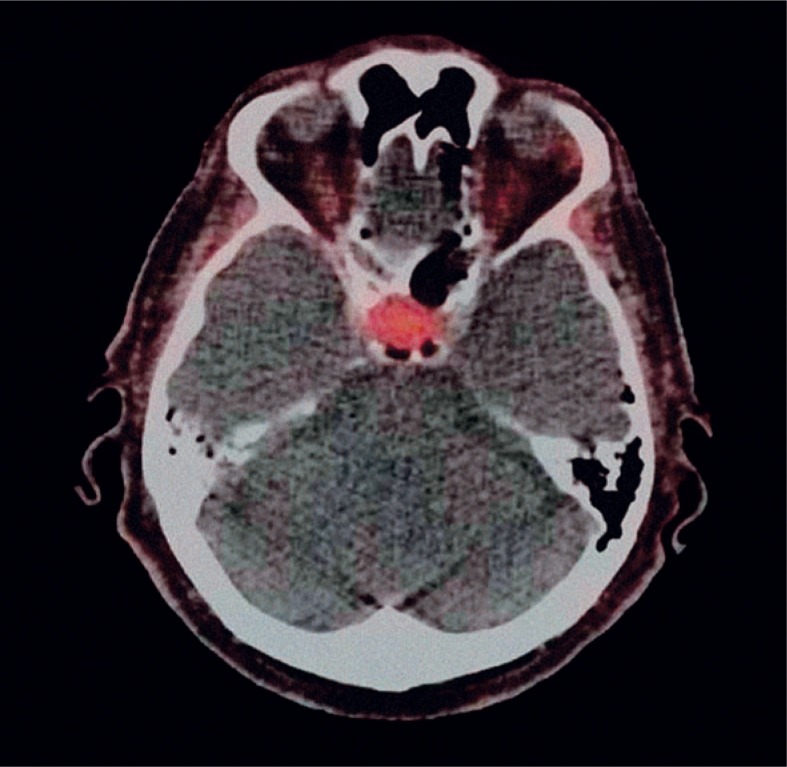
Positron emission tomography-computed tomography axial slice: Macroadenoma of pituitary gland that incidentally took-up ^18^F-fluorocholine (SUVmax 3.7).

**FIGURE 2.**
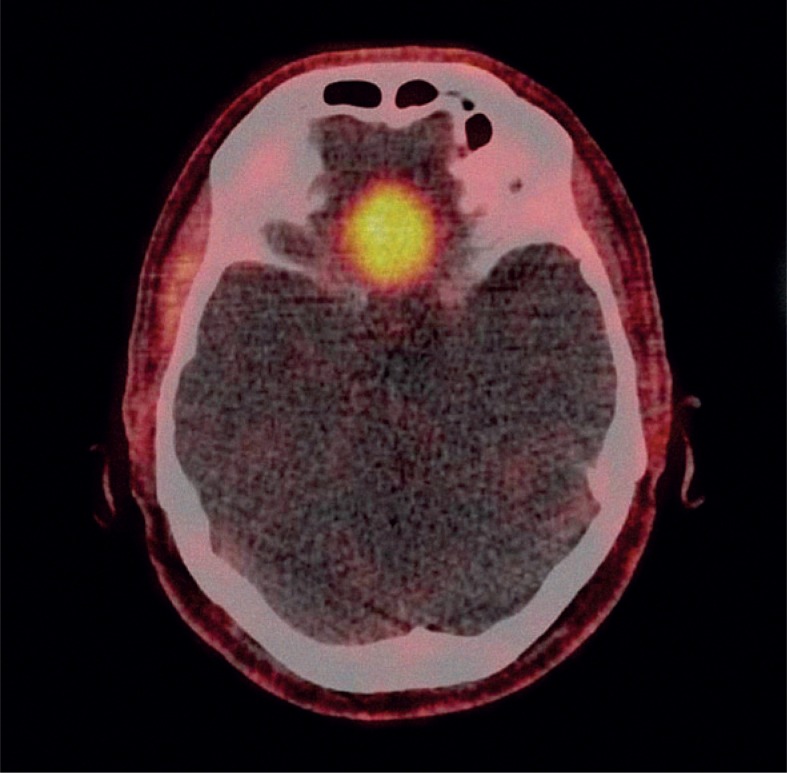
Positron emission tomography-computed tomography axial slice: Meningioma in the anterior cranial fossa that incidentally took-up ^18^F-fluorocholine (SUVmax 3.7).

**FIGURE 3.**
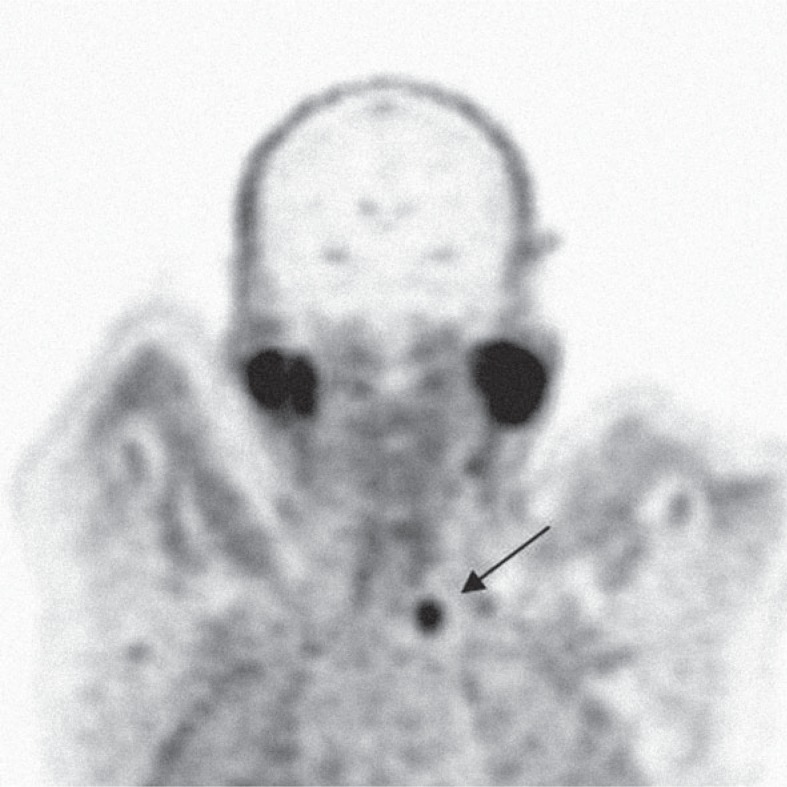
Positron emission tomography-computed tomography coronal slice: Adenoma of the parathyroid that incidentally took-up ^18^F-fluorocholine (SUVmax 3.4).

**FIGURE 4.**
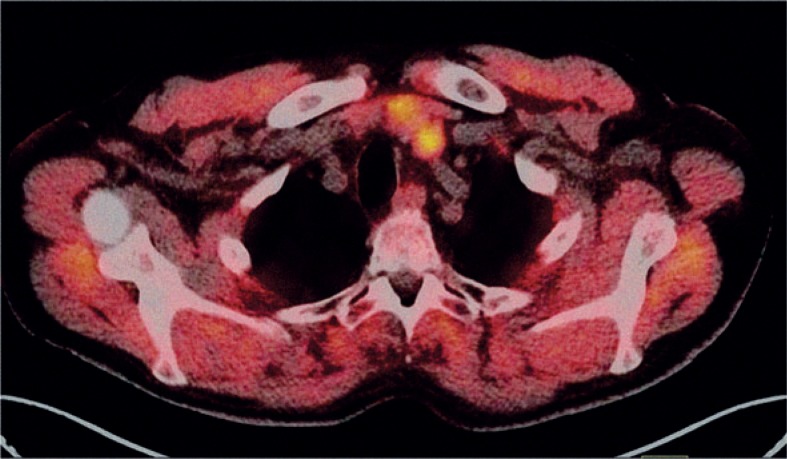
Positron emission tomography-computed tomography axial slice: Thyroid adenoma that incidentally took-up ^18^F-fluorocholine (SUVmax 3.3).

In 8 patients referred to FCH PET/CT for prostate cancer, an incidental focus was found in the head and neck region, which was finally diagnosed as corresponding to a benign tumour (Table 1). The standard of truth was histology in 2 cases, correlative imaging with MRI in 4 cases, 99mTc-sestaMIBI scintigraphy, ultrasonography and biology in one case, biology including PTH assay in one case. There were 3 pituitary adenomas (Figure 1), 2 meningiomas (Figure 2), 2 hyperfunctioning parathyroid glands (Figure 3) and 1 thyroid adenoma (Figure 4).

In 2 patients, the incidental focus corresponded histologically to a malignant lesion: 1 papillary carcinoma of the thyroid 45 mm in size and 1 cerebellar metastasis of a second primary cancer in the lung.

## Discussion

To the best of our knowledge, the frequency of incidentalomas discovered on FCH PET/CT in head or in the neck has never been published. It was estimated to be 1.9% in our series, a thyroid nodule being the most frequent cause of incidentaloma (41%). Actually, our survey showed that the mention in the report of an incidental FCH uptake in the head or the neck led to further investigations in only 52% of cases. The variable impact of this discovery on patient management is probably due to the fact that, in patients with known prostate cancer, the characterisation of a brain lesion was considered more important than that of thyroid nodule or a focus in the thyroid bed. Overall the final diagnosis of those incidental foci which were further explored corresponded to a non-malignant lesion in 80% of cases (8/10).

The main limitation of this study is the fact that not all incidentalomas have been explored and that the final diagnosis of benign tumour is based on histology in only two cases (Table 1): one pituitary adenoma (patient #3) and one thyroid adenoma (patient #8). In the other cases, it has been set by a multidisciplinary medical team on follow-up data which were independent from FCH PET/CT.

Providing that those preliminary findings would be confirmed in larger series, we will briefly discuss if deliberately performing FCH PET/CT in patients presenting with or suspected of a benign tumour in the head or in the neck could be justified.

### Pituitary adenomas

The pituitary gland has a moderately intense physiologic uptake of FCH, as shown on PET/MRI^7^, although it has been neglected in some previous articles using PET/CT.^5^ In the 3 cases of pituitary adenoma of our series, an intense tumour uptake was observed.

To the best of our knowledge, this is the first mention of this incidental finding on FCH PET/CT. In one case, unexpected recurrence of a resected adenoma, suspected on FCH PET/CT, was confirmed by MRI. An inflammatory reaction after the surgical resection cannot be ruled out but seems improbable after three years. In another case, a non-functioning pituitary adenoma was confirmed at surgery; the patient also had another incidental FCH focus in the left vocal cord which was demonstrated to correspond to a calcified granuloma, confirming that inflammatory head and neck lesions can also show FCH uptake.^5^

The FDG uptake by functioning and non-functioning pituitary adenomas had been documented by several case reports and one series of such cases.^8^ FDG pituitary focus corresponded in most cases to macroadenoma and only rarely to microadenoma or malignancy.^9^ However, in case of adrenocorticotrophic hormone (ACTH) or growth hormone (GH) producing microadenomas, FDG PET, as a complement to MRI, resulted in 12 positive readings of 20 surgically verified pituitary microadenomas.^10^ The potential superiority of aminoacid PET tracers 11C-tyrosine and 11C-methionine over FDG in the visualisation and therapy follow-up of pituitary adenomas, in particular ACTH-secreting adenomas has been demonstrated.^11^–^13^ PET imaging of prolactinomas and GH-secreting adenomas with 11C-raclopride, a D2 radioligand, was proposed in the differential diagnosis with meningiomas and skull base neuromas and in treatment monitoring.^14^ Somatostatin receptors (SSR) can also be over-expressed in functioning pituitary adenomas, in particular those which are ACTH-secreting. On the other hand, no incidental uptake by a pituitary adenoma on SSR PET/CT has been reported until now, probably due to the physiologic pituitary uptake of the somatostatin analogue labelled with ^68^Ga. In contrast SSR-PET/CT performed in a deliberate search for the source of inappropriate ACTH serum levels may lead in rare cases to detecting a pituitary adenoma in an unexpected location.^15^–^16^

We conclude from our observations that a definite FCH uptake in the pituitary should lead to characterisation of a probable lesion, at least with MRI. If recurrence or persistence of a known pituitary adenoma is suspected, it is doubtful whether FCH PET/CT will have any indication, or whether FDG should be preferred if PET/CT is indicated and 11C-labeled tracers not available. Similarly to 11C-methionine and in contrast with FDG, FCH has the advantage of a low background activity in the brain cortex.

### Meningiomas

FCH uptake is faint in the normal brain parenchyma, which permits PET/CT detection of gliomas, even in some low grade tumours, and also of meningiomas^5^, as illustrated in the present study. It has been suggested that dynamic FCH PET acquisition can differentiate between those tumours.^17^ PET/MRI may also be useful to distinguish between glioblastoma and meningioma that both showed moderately intense FCH uptake while it was faint in brain tumours of a lower grade.^7^

Detection of meningiomas with ^11^C-choline PET/CT was also reported.^18^ Relative to the contralateral side, 11C-choline uptake was increased in all 7 meningiomas, whereas FDG uptake was decreased in 6 patients and increased in 1 of the 2 patients with grade II meningiomas. 11C-acetate, another lipid PET tracer, showed high uptake in all 20 meningiomas, in contrast to the low uptake in surrounding normal brain tissue^19^, whereas with FDG 17 foci appeared photopenic and 3 hyper-intense. 13N-ammonia also had relatively greater uptake in 10 meningiomas when compared with FDG.^20^ Aminoacid PET tracers are also capable of demonstrating meningiomas: for delineation of gross tumour volume in stereotactic radiotherapy using ^11^C-methionine^21^, or in recurrent cases using ^18^F-fluoroethyltyrosine^22^, or in one patient referred to FDOPA PET for Parkinson’s disease.^23^ Furthermore, imaging meningioma with SSR scintigraphy has been reported for more than two decades and, more recently, a potential role for SSR PET/CT has been assessed, the detection rate being better than that of MRI.^24^

In conclusion, an intense incidental FCH uptake may lead to discovery of a meningioma; in prostate cancer patients, this can have a major impact on their management since anti-androgen therapy would favour tumour development and put them at risk for neurological symptoms. It is clear that the field of view of the “whole-body” FCH PET/CT acquisition should include the brain^6^ in case of prostate cancer. In case of a known meningioma, determination of metabolic volume prior to radiotherapy or for surgery guidance is a valid indication for PET imaging. However many tracers can be used; for the moment, FCH may be seen as a newcomer.

### Hyperfunctioning parathyroid glands

To the best of our knowledge, FCH uptake by hyperfunctioning parathyroid glands has never been described before. In one recent case report, a parathyroid adenoma was discovered on ^11^C-choline PET/CT.^25^ The discovery of an incidental hyper-functioning parathyroid gland on FDG PET has been reported.^26^–^27^ FDG has been proposed to stage parathyroid carcinoma, being more aggressive than adenoma or hyperplasia.^28^ Discrepant results have been reported with FDG in the detection of adenoma or hyperplasia, a very low sensitivity in two short series^29^,^30^ or, in comparison with ^99m^Tc-SestaMIBI, a better sensitivity (86% *vs*. 43%) but a lower specificity (78% *vs*. 90%)^31^; no recent series have been published. ^11^C-methionine is currently the PET competitor for the detection of parathyroid adenomas, with a patient-based sensitivity of 81% and specificity of 70% according to a recent meta-analysis.^32^

In conclusion, in the case of patient #6, unexpected parathyroid tumours can be localised on FCH PET/CT, on basis of the anatomical location on CT of a cervical FCH focus. Such an incidental image should prompt biochemical work-up, including serum PTH assay (patient #7), as prolonged hyperparathyroidism will be detrimental in those elderly patients and requires medical or surgical treatment.

Whether localising hyperfunctioning parathyroid glands could become an indication for FCH PET/CT requires comparative studies *vs*. the reference functional imaging ^99m^Tc-SestaMIBI/^123^I scintigraphy and/or vs. ^11^C-methionine, the PET competitor. FCH PET imaging will benefit from a better resolution than SPECT, and delivery will be easier than for ^11^C-methionine. But differential diagnosis with thyroid nodules taking-up FCH, as illustrated in our series, will be difficult, in particular in case of multinodular goitre, since dual isotope acquisition is not possible and subtraction technique will be very difficult.

### Thyroid adenomas

Concerning FCH uptake by a benign thyroid nodule, two cases similar to that of patient #8 have been reported recently.^33^,^34^ Numerous articles addressed the diagnostic value of incidental FDG uptake by a thyroid nodule. It actually corresponds to a non-malignant origin in a majority of cases, 70%, 59% or 65% as derived from two recent large series and a meta-analysis.^35^–^37^

The FCH foci in the thyroid gland previously reported in literature corresponded to benign adenomas.^33^,^34^ However, in the present series 1 of the 2 incidental thyroid foci of FCH uptake which could be characterised corresponded to a papillary carcinoma. ^11^C-choline has even been proposed in the detection of thyroid carcinoma and its metastases, performing better than FDG in a preliminary series of 4 patients.^38^ Therefore, it seems prudent that FCH uptake by a thyroid nodule prompts an adequate work-up to rule-out thyroid cancer.

In contrast it is unlikely that FCH may helpful in characterising a thyroid nodule as malignant or benign. Even FDG lacks specificity for the detection of cancer in a thyroid nodule and, since FCH usually detects less-aggressive tumours, it may be expected non-malignant nodules would take-up FCH even more frequently.

### Cost and cost-effectiveness

In the present study, the detection of those incidental lesions per se implied no extra-cost since FCH PET/CT was indicated because of prostate cancer. In the above discussion, we then speculated that the present finding of FCH uptake in some of those incidentalomas could lead to a deliberate indication of FCH PET/CT in patients with a diagnosed or suspected benign tumour. In this case, the cost-effectiveness of FCH PET/CT should be re-evaluated for each type of benign tumour, bearing in mind the cost of FCH PET/CT and of the alternative nuclear medicine examinations.^39^ Briefly, PET/CT with FCH is more expensive than with FDG, and PET/CT is usually more expensive than SPECT/CT due to the higher price of the machine, but this is not always true *e.g*. 111In-pentretrotide, somatostatin receptor ligand for SPECT, one of the alternatives for meningioma detection, is more expensive than FDG or FCH.

## Conclusions

We described incidental FCH uptake in the head or in the neck, in 1.9% of the PET/CTs performed for staging or restaging prostate cancer. Some of those incidental FCH foci corresponded to malignancies, but more frequently (80%) to various benign tumours. In particular, for the first time, we observed FCH uptake in pituitary adenomas and in hyperfunctioning parathyroid glands. Such foci should be mentioned in the report, as meningioma or hyperparathyroidism may directly impact on management of a patient with prostate cancer. Since FCH is taken-up by slow-growing malignancies it could be expected that FCH PET/CT can detect benign tumours even more frequently than FDG PET/CT.

Furthermore, there might be a future indication for FCH PET/CT when one such tumour is already known or suspected: for post-operative control of a resected pituitary adenoma, to guide surgery or radiotherapy of a meningioma or to localise hyper-functioning parathyroid glands. In those indications, comparative studies with reference PET tracers could be undertaken, on basis of published case reports and the present preliminary series.

## Figures and Tables

**TABLE 1.** Patients with incidentaloma in the head or in the neck on FCH PET/CT performed for prostate cancer staging or restaging benign tumours

**No.**	**Age**	**Prostate cancer setting for FCH PET/CT**	**Incidental FCH uptake in head and neck region**	**Diagnostic modality(ies) for characterisation**
**1.**	78	Biochemical recurrence under HT	Pituitary	MRI: Macroadenoma of pituitary gland
**2.**	64	Biochemical recurrence after HT	Pituitary	MRI: Residual pituitary adenoma in the right side of sella turcica
**3.**	81	Biochemical recurrence after prostatectomy, under HT	Pituitary	Post-surgical histology: non-functioning pituitary adenoma
**4.**	72	Initial staging	Frontal lobe	MRI: Meningioma in the anterior cranial fossa
**5.**	70	Biochemical recurrence, under HT	Between cerebellum and medulla	MRI: Meningioma at the level of foramen magnum on the right side
**6.**	56	Initial staging	Behind the left thyroid lobe	Serum PTH: 160 ng/L, ultrasonography and 99mTc-SestaMIBI/123I scintigraphy: recurrent parathyroid adenoma at the same location
**7.**	75	Biochemical recurrence after high intensity focused ultrasound, no HT	Behind the left thyroid lobe	Serum PTH: 134 ng/L, calcemia: 2.6 mmol/L, normal serum calcidiol
**8.**	52	Biochemical recurrence after prostatectomy, under HT	Left thyroid lobe	Ultrasonography: multinodular thyroid gland. Cytology of the target nodule: thyroid adenoma

HT = hormonal treatment; PTH = parathyroid hormone; MRI = magnetic resonance imaging
